# miR-21/SMAD2 Is Involved in the Decrease in Progesterone Synthesis Caused by Lipopolysaccharide Exposure in Follicular Granulosa Cells of Laying Goose

**DOI:** 10.3390/metabo14070362

**Published:** 2024-06-27

**Authors:** Xinyi Guo, Shijia Ying, Huiping Xiao, Hao An, Rihong Guo, Zichun Dai, Wenda Wu

**Affiliations:** 1 MOE Joint International Research Laboratory of Animal Health and Food Safety, College of Veterinary Medicine, Nanjing Agricultural University, Nanjing 210095, China; 2021107103@stu.njau.edu.cn (X.G.); ysj@jaas.ac.cn (S.Y.); 2021807175@stu.njau.edu.cn (H.X.); 2019807162@njau.edu.cn (H.A.); 2 School of Food and Biological Engineering, Hefei University of Technology, Hefei 230009, China; 3 Key Laboratory for Crop and Animal Integrated Farming, Ministry of Agriculture and Rural Affairs, Institute of Animal Science, Jiangsu Academy of Agricultural Sciences, Nanjing 210014, China; rhguo@jaas.ac.cn (R.G.); 20210064@jaas.ac.cn (Z.D.)

**Keywords:** lipopolysaccharide, granulosa cells, miR-21, progesterone, goose

## Abstract

Lipopolysaccharide (LPS) is one of the important pathogenic substances of *E. coli* and *Salmonella*, which causes injury to the reproductive system. Ovarian dysfunction due to Gram-negative bacterial infections is a major cause of reduced reproductive performance in geese. However, the specific molecular mechanisms of LPS-induced impairment of sex steroid hormone synthesis have not been determined. The regulatory mechanism of miRNA has been proposed in many physiological and pathogenic mechanisms. Therefore, the role of miRNA in breeding geese exposed to LPS during the peak laying period was investigated. In this study, twenty Yangzhou geese at peak laying period were injected with LPS for 0 h, 24 h, and 36 h. The follicular granulosa layer was taken for RNA-seq and analyzed for differentially expressed miRNAs. It was observed that LPS changed the appearance of hierarchical follicles. miRNA sequencing analysis was applied, and miR-21 and SMAD2 (SMAD family member 2) were selected from 51 differentially expressed miRNAs through bioinformatics prediction. The results showed that miR-21 down-regulated SMAD2 expression and progesterone (P4) production in LPS-treated goose granulosa cells (GCs). It also determined that overexpression of miR-21 or silence of SMAD2 suppressed the sex steroid biosynthesis pathway by decreasing STAR and CYP11A1 expression. Down-regulation of miR-21 exacerbates the LPS-induced decline in P4 synthesis and vice versa. The findings indicated that miR-21 was involved in LPS regulation of P4 synthesis in goose granulosa cells by down-regulating SMAD2. This study provides theoretical support for the prevention of LPS-induced ovarian dysfunction in geese.

## 1. Introduction

Egg-laying performance of geese depends on the function of the ovaries and the follicles development ability [1]. Ovarian Function includes Reproductive and endocrine functions. Considerable research has indicated that steroid hormones synthesis in ovary follicular granulosa cells (GCs) is crucial for the regulation of folliculogenesis, follicular development, maturation, and atresia [2].

The ovaries of birds are slightly different from those of mammals in that follicular development in poultry has a strict hierarchical arrangement [3]. Their developmental capacity is regulated by a complex and subtle network, with the hypothalamic–pituitary–gonadal (HPG) axis playing an essential role in follicular development and maturation through the regulation of the synthesis of a wide range of steroid hormones [4]. The ovary has endocrine functions. The ovary of a bird consists of follicles in various stages of development. During the breeding season, the yolk-filled follicles are arranged in a hierarchy. It contains a large number of pre-hierarchical follicles and 3–5 hierarchical follicles, and it is significantly correlated with egg production [5]. Another determinant of egg production performance is progesterone (P4) [6]. It is one of the steroid hormones secreted by the granular layer of the avian hierarchical follicle and plays a decisive role in follicular development and ovulation. Genes such as steroidogenic acute regulatory protein (STAR), cytochrome P450 family 11 subfamily A member 1 (CYP11A1), and 3β-hydroxysteroid dehydrogenase (HSD3B1) are directly involved in P4 synthesis [6,7]. Furthermore, transcription factors such as GATA-4/6, CREB, SP-1, SMAD2/3, and regulatory factors (IGF-1, TGF-β, SF-1) regulate ovarian steroid synthesis and gene expression through activation of intracellular signaling pathways [8,9].

Lipopolysaccharide (LPS), a component of the cell wall of Gram-negative bacteria such as *E.coli* and *Salmonella*, is widely used as a reagent for inflammatory modeling [10]. LPS activates cellular signaling through TLR4 and triggers an inflammatory response while causing a series of adverse effects [11]. Immune system challenges caused by LPS will affect physiological and biochemical processes and interfere with the endocrine system and hormone levels in animals. The reproductive toxicity of LPS is well established [12,13]. Especially in poultry breeding, poor animal management and intensive breeding lead to LPS exposure, which may result in reduced productivity and economic losses. LPS exposure led to the disorder of sex steroids, luteolysis, and follicular atresia in mammals. It has been shown that intrauterine infusion of LPS can lead to premature luteolysis in heifers [14]. Furthermore, long-term exposure to LPS promotes ovarian granulosa cell pyroptotic death and ovarian interstitial cell fibrosis in mice [15]. In birds, studies have shown that high concentrations of LPS in the environment and blood can affect follicular development and production performances [16]. Meanwhile, changes in the concentration of various sex hormones, such as P4 and estradiol, are often presented as evidence that LPS affects ovarian function, fully demonstrating the correlation between LPS and sex steroid hormone secretion processes.

The laboratory previously designed an anti-seasonal feeding model for Yangzhou geese that allowed breeding geese to lay eggs from May to September, which improved the farmers’ income and balanced the year-round production, a novel and unique efficiency model [17]. However, the problem of LPS pollution caused by modern intensive farming is more severe in the hot and humid summer. Due to the cloacal structure of birds, the ovaries are the preferred infective tissue for *E. coli* and *Salmonella*. P4 synthesis in avian pre-hierarchical follicular GCs is more susceptible to LPS [18]. A study has shown that *Salmonella* inhibited granulosa cells development and reduced egg production in chickens, a process mediated by down-regulation of CYP11A1 expression and modified to STAR and SMAD family member 2 (SMAD2) [19]. Hitherto, most studies on reproductive performance have focused on membrane signaling pathways, and the molecular mechanisms by which LPS affects P4 synthesis in goose GCs have yet to be reported.

miRNAs have crucial roles in various aspects of ovary function, including steroidogenesis, follicular development, ovulation, and gene regulation in poultry. MicroRNAs (miRNAs) are approximately 22 nucleotides long, small endogenous RNAs that function in post-transcriptional gene silencing by binding to mRNA 3′ UTR [20]. MiRNAs are widely distributed in different cells and tissues and are involved in a variety of biophysiological processes, playing an integral regulatory role in the pathogenesis of disease, tumors, and cancer. miRNAs can participate in almost all life activities. A large number of miRNAs are present in animal ovaries involved in reproductive regulation, and multiple groups of miRNAs are differentially expressed during follicle development and in different injury models of ovarian type [21]. Studies suggest that miRNAs have a role in regulating luteinizing steroid production in heifers [22]. Currently, GCs are the primary focus of research on miRNA regulation and ovarian function in mammals. Numerous studies have demonstrated the significant role of miRNAs in regulating follicular development and atresia, cell proliferation and apoptosis, steroid hormone synthesis, and even ovarian cancer. In mammals, miR-320a, miR-323-3p, miR-383, miR-1246, miR-31, and miR-20b have been found to influence sex steroid hormone synthesis in GCs [23,24,25,26,27]. In poultry, however, the study of functional miRNAs in ovarian function is still limited. Only a few studies have demonstrated the significance of miR-199-3p, miR-133a-3p, and miR-181a-5p in regulating follicular development and hormone secretion in geese [28,29,30].

In summary, LPS readily contaminates the avian ovary and affects P4 synthesis. miRNA is involved in the occurrence and development of reproductive system injury. The abnormal expression of miRNA in many groups is related to follicular atresia and granulosa cells injury. To investigate whether the effect of LPS exposure on P4 synthesis is regulated by miRNA in goose GCs, in the present study, a second-generation sequencing strategy was applied to analyze miRNAs and mRNAs in goose GCs from hierarchical follicles exposed to LPS. The miR-21 in the goose was described for the first time, and the expression level was determined. Differently expressed target genes and miRNAs were screened with the aim of identifying potential signaling mechanisms. The miRNA target genes were predicted by bioinformatic methods, and the impacts of miR-21 overexpression or suppression on P4 synthesis were studied in the goose primary GCs. Furthermore, the regulatory function of miR-21 in goose primary GCs was verified. The findings can contribute to a better understanding of the mechanism of miR-21 involved in the decrease in P4 synthesis induced by LPS in goose GCs.

## 2. Materials and Methods

### 2.1. Ethics Statement

Twenty Yangzhou geese raised at the Liuhe experimental farm were used in this study. The animals were allowed access to feed and water ad libitum under normal conditions and were executed by jugular vein bleeding to minimize suffering. The experimental procedures were approved by the Research Committee of Jiangsu Academy of Agricultural Sciences and conducted with adherence to the Regulations for the Administration of Affairs Concerning Experimental Animals (Decree No. 63 of the Jiangsu Academy of Agricultural Science on 8 July 2014).

### 2.2. Animals and Treatments

Yangzhou geese of about 360 days (bodyweight 7.5–8 kg) at peak laying period were raised under natural photoperiod to eat and drink ad libitum. The behavior of laying is monitored by computer video. Twenty geese were exposed to LPS from *E. coli* 055: B5 (Sigma, Shanghai, China) for 0 h, 24 h, and 36 h by wing intravenous injection: ten animals in the control group and five animals in each of the treatment groups. Based on preliminary laboratory studies and pre-experiments [18], the LPS treatment concentration was determined to be 1.5 mg/kg BW. The egg-laying time of each goose was recorded, and LPS was treated at −28 h, −16 h, and 8 h relative to egg-laying time to ensure that LPS was applied to the ovulation period. Geese were slaughtered and sampled promptly 8 h after laying.

### 2.3. Sample Collection and RNA Isolation

Before slaughter, blood samples (2 mL) were collected by wing vein puncture. The serum was separated by centrifugation at 4 °C and stored at −20 °C until assay. The preovulatory follicles of the hierarchy are identified according to size, with the F1 follicle being the largest follicle and the next follicle to ovulate, followed by the F2 follicle, the second-largest follicle, etc. The granulosa layer of the hierarchical follicles (F1–F5) was isolated and snap-frozen in liquid nitrogen. Both the GCs of hierarchical follicles of normal appearance and the GCs of the denatured hierarchical follicles were collected and snap-frozen in liquid nitrogen, then stored at −80 °C for further experiments. TRIzol reagent was used to extract total RNA from follicles tissues and GCs, according to the manufacturer’s instructions.

### 2.4. miRNA and mRNA Sequencing

cDNA libraries were constructed using the NEB Next Ultra-small RNA Sample Library Prep Kit for Illumina (New England Biolabs, Ipswich, MA, USA). The concentration of RNA samples was accurately quantified by Qubit 2.0, the purity of RNA samples was checked by Nanodrop (Thermo Fisher, Waltham, MA, USA), and RNA sequencing of mRNA and miRNA was performed by Illumina HiSeq X Ten (Illumina, San Diego, CA, USA). The reference genome sequence was Goose V1.0 (GCA_002166845.1). Differential expression analysis was performed using the edgeR R package. EdgeR (v3.8.6) provides statistical routines for determining differential expression in digital miRNA expression data using a model based on the negative binomial distribution. miRNA with a *p* < 0.01 found by DESeq (v1.18.0) were assigned as differentially expressed. The target genes of miRNAs were filtered using miRanda (v3.3a) with the parameters Score ≥ 140, Energy ≤ −1.0. The expression of miRNA was counted and normalized using the TPM method.

$$TPM=\frac{Readcount*1,000,000}{MappedReads}$$

### 2.5. Cell Culture and Transfection

Goose hierarchical follicle granulosa cells were used in this experiment. After the ovaries were taken out, the F1–F5 follicles were separated and placed in 4 °C saline, the vascular and basement membrane layers were stripped, the follicles were cut open so that the yolk was released into saline, and the white translucent granular layer floating on the yolk was carefully stripped. Cells were cultured in a humidified atmosphere containing 5% CO_2_ and 95% air at 37 °C in complete medium consisting of 89% M199 cell culture medium, 10% fetal bovine serum, and 1% penicillin-streptomycin. Primary GCs (4 × 10^5^ cells/well in 1 mL of medium) were cultured in 12-well plates. When the cell density reached 70%, GCs were treated with LPS (1 μg/mL) for 12 h. Cells were collected by TRIzol, and the concentration of P4 was detected using an ELISA kit. To further explore the mechanism of miR-21-involved LPS-induced P4 secretion decrease, transfection experiments were performed in GCs.

The RNA oligonucleotides were designed according to the sequences of miR-21 (miRBase accession number: MIMAT0021865). Among them, miR-21 inhibitor, miR-21 mimics, siSMAD2, and Negative Control were constructed by GenePharma (Shanghai, China). Cells were transfected with 50 nM of miR-21 inhibitor, miR-21 mimics, and siRNA targeting goose SMAD2 using Lipofectamine 3000 (Invitrogen, Shanghai, China). The non-targeting pool Negative Control was used as transfection control.

### 2.6. Quantitative Real-Time PCR

After treatments, total RNA was extracted. The mRNA was reverse-transcribed using Prime Script RT Master Mix Perfect Real Time, whereas miRNA was reverse-transcribed using the one-step miRNA cDNA synthesis kit. The real-time qPCR was performed in triplicate per sample.

qRT-PCR was performed on an ABI 7500 system (Applied Biosystems, Shanghai, China) using FastStARt Universal SYBR Green Master Mix. The β-actin gene was used as the reference gene; all qRT-PCR gene-specific primers were designed using Oligo 7.0. The primer sequences are presented in Table S1. Relative gene expression levels were calculated using the 2^−∆∆CT^ method with a significance level of *p* ≤ 0.05.

### 2.7. ELISA

Following the treatments, the cell culture medium was immediately assayed for P4 levels using a competitive enzyme immunoassay kit (Beijing North Institute of Biological Technology, Beijing, China) according to the manufacturer’s instructions. After treatment, culture medium was collected for P4 ELISA analysis, and the cells were collected for DNA extraction. The final P4 levels were analyzed using the following equation, as described by Silva et al. [31]: P4 levels ng/mL/ng DNA = P4 concentration in culture medium/total amount of DNA. Each sample was measured three times, and three separate experiments were conducted.

### 2.8. Statistical Analysis

Statistical analyses were performed using the IBM SPSS Statistics program (version 26). Normality of the data was tested using the Shapiro–Wilk normality test. Nonparametric data with multiple comparisons were analyzed by Kruskal–Wallis test (including Dunn–Bonferroni post hoc correction). The Mann–Whitney U test was used for comparison between two groups. Data with normal distribution were analyzed by one-way ANOVA with Tukey’s correction for multiple comparisons, and the Student *t* test was used for comparison between two groups. Values are presented as the Mean ± Standard Error of the Mean (Mean ± SEM). All tests were two-sided, and data were considered statistically significant if *p* values ≤ 0.05. Graphs were prepared in GraphPad Prism (version 8.2.1). *, *p* < 0.05; **, *p* < 0.01. Ns, not significant.

## 3. Results

### 3.1. Morphological Observation and Reproductive Hormone Levels

**Figure 1:**
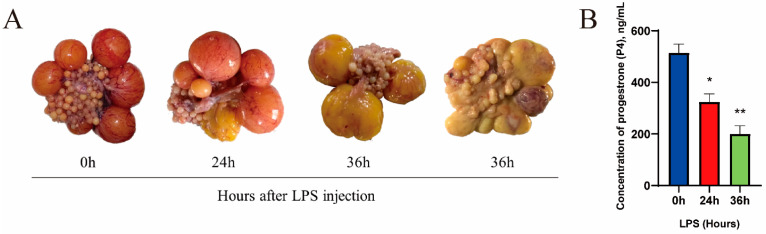
Comparison of morphological characteristics and reproductive hormone levels of HFs and DFs. (**A**) Morphological observation of goose ovarian follicles after 0 h, 24 h, and 36 h treated with LPS. (**B**) reproductive hormone progesterone levels in HF and DF (*n* = 3). Data are presented as mean ± SEM. *, *p* < 0.05; **, *p* < 0.01.

Ovarian morphology was observed and photographed, and it was found that follicles at different stages of development were present in the ovaries of geese; the morphology of hierarchical follicles changed considerably with increasing LPS treatment time. No significant changes in the morphology of the hierarchical follicles were observed after LPS treatment for 0 h. After LPS treatment for 24 h, the hierarchical follicles in some geese (3/5) became deep yellow in appearance and irregular ellipses or were round. After LPS treatment for 36 h, the hierarchical follicles of all geese (5/5) turned dark yellow and were irregularly elliptical in appearance, and the yolk became gelatinous (Figure 1A). This resulted in difficulty in separating the follicular membrane layer from the granular layer. Interestingly, there was no significant change in the appearance of pre-hierarchical follicles during this process, indicating that the response of hierarchical follicles to LPS stimulation was much higher than that of pre-hierarchical follicles. In addition, serum P4 concentrations were significantly higher in geese with normal follicular appearance than in geese with degenerated follicles and negatively correlated with the time of LPS treatment (Figure 1B).

Based on the above findings, the GCs of normal appearance (LPS treatment for 0 h, HF) and the GCs of the denatured hierarchical follicles (LPS treatment for 36 h, DF) were collected for miRNA sequencing with three biological replicates per group.

### 3.2. miRNA RNA-Seq Profiling

**Figure 2:**
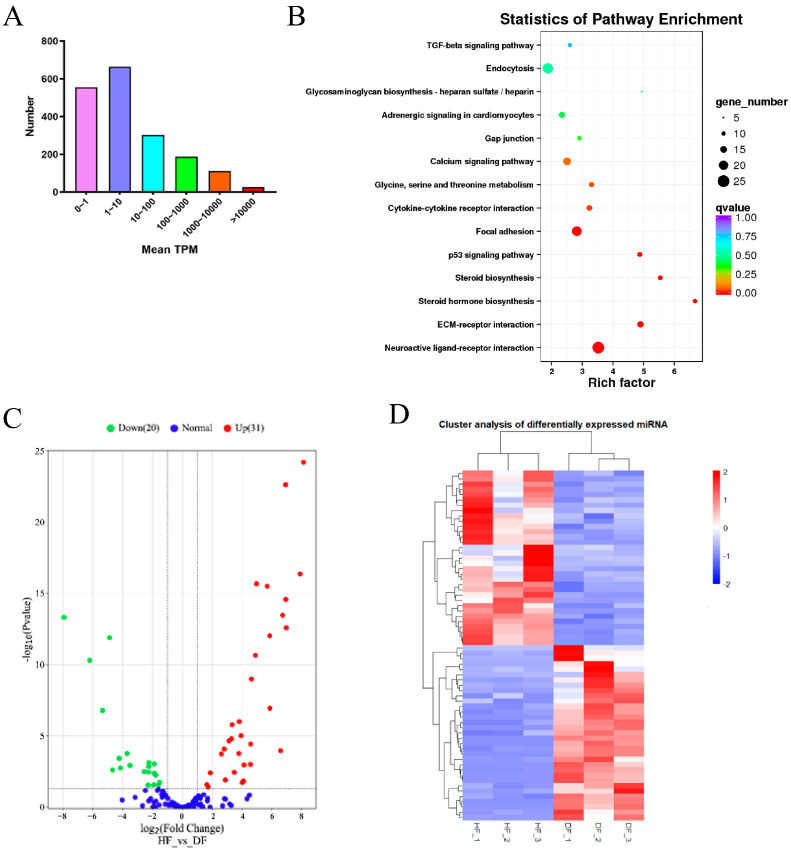
Overview of the miRNA RNA-seq data. (**A**) Distribution of miRNAs with 0 to >10,000 mean transcripts per million (TPM). (**B**) KEGG analyses of the target genes of differentially expressed miRNAs. (**C**) Volcano map of differentially expressed miRNA. (**D**) Clustering heat map of differentially expressed miRNAs.

All the miRNA sequencing data are deposited in the SRA database with the accession number PRJNA 916249. Small RNA sequencing yielded between 17 M and 30 M raw reads in the six samples. The sequencing quality values Q30 were all greater than 98%. After the low-quality reads with connectors were removed, the clean reads obtained were greater than 79% (s). Most of the clean reads were between 21 and 23 nt in length, and read counts with 22 nt were the most abundant. Of note, 834 known miRNAs were obtained by comparison, and 1016 unknown miRNAs were discovered. Based on the Transcripts Per Kilobase of exon model per Million mapped reads (TPM), miRNA expression levels were divided into different intervals. Most miRNAs were distributed between 1 and 10 TPM, and only a few miRNAs were of high abundance (>100 TPM; Figure 2A). In general, the number of miRNAs was negatively correlated with the value of TPM, indicating that that only a few miRNAs could affect follicular P4 secretion. Analysis of the transcriptome sequencing quality data from the control group (HF) and LPS-treated group (DF) revealed that the Q30 of each group of samples was higher than 98.56% (Table S2).

### 3.3. Differentially Expressed miRNAs between HF and DF

A total of 51 differentially expressed miRNAs (DEMs) were identified among the known miRNAs, of which 31 miRNAs were highly expressed in the healthy follicles compared to the atretic follicles, whereas the other 20 miRNAs were down-regulated in the healthy follicles compared to the atretic follicles (Figure 2B). Cluster analysis showed that miRNA expression patterns were different in HFs and DFs (Figure 2C). To better understand the biological functions of the 51 DEMs identified, the potential target genes of miRNAs were predicted. This returned 3782 terms related to target genes, including 2444 unique genes. Subsequent miRNA-targeted pathway union analysis revealed 60 KEGG pathways significantly (*p* < 0.05) related to genes targeted by up-regulated or down-regulated miRNAs. Several pathways were involved in follicle and corpus luteum development, including the PI3K-Akt signaling pathway, steroid biosynthesis, apoptosis, and the Hippo signaling pathway (Figure 2D).

The results of GO enrichment analysis showed that the enrichment of DEM target genes in biological process mainly involves cellular processes, developmental processes, reproductive process, and reproduction (Figure S1).

### 3.4. Functional Analysis of DEMs

**Figure 3:**
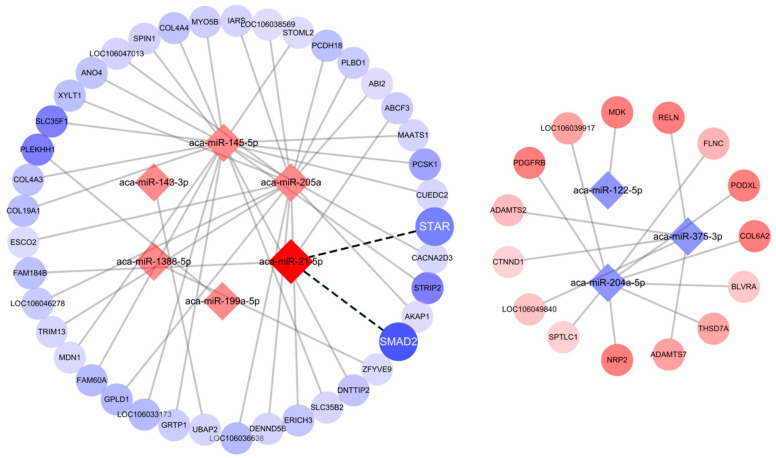
Construction of a miRNA-gene pathway regulatory network.

**Figure 4:**
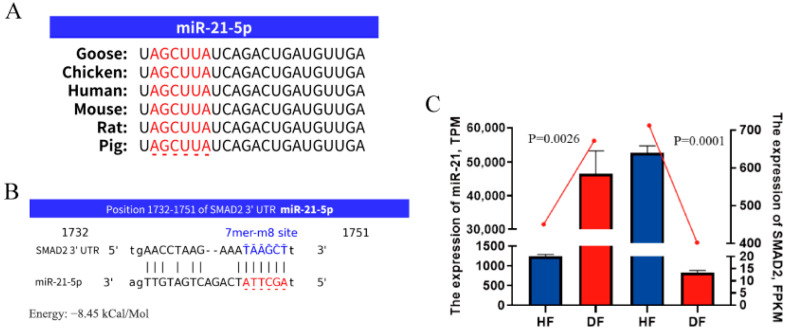
SMAD2 mRNA were predicted as target genes of miR-21. (**A**) Comparison of miR-21 initial sequences (marked in red) of different species. (**B**) Highly conserved 7mer-m8 binding site (marked in blue) of miR-21 and SMAD2 among multiple organisms were predicted by miRanda. (**C**) Comparison of expression levels of miR-21 and SMAD2 in the HF and DF RNA sequencing. Data are presented as mean ± SEM, n = 3.

To investigate the mechanism of miRNA involvement of LPS on P4 secretion in GCs, ten highly expressed DEMs were selected as candidate genes for biological function validation (Table 1). Target gene prediction was performed using the bioinformatics prediction software miRDB and miRanda (Table S3). In addition, mRNA transcriptome sequencing was performed, and the target genes were predicted. The miRNA–mRNA interaction networks were constructed to identify their specific biological functions (Figure 3). The findings indicated that miR-21 initial sequences is a widely conserved aspect in human, mouse, rat, chicken, and pig (Figure 4A), and miR-21 has a 7mer-m8 conserved binding site with goose SMAD2 (Figure 4B). miRNA and mRNA sequencing results showed that miR-21 expression was higher in HF than DF, while the target genes SMAD2 (Figure 4C) showed a contrasting trend with higher miR-21 expression. This result is consistent with the expression pattern of miRNAs. Combined with KEGG in Figure 2D, the steroid hormone synthesis-related pathway was enriched. Therefore, miR-21 was selected for functional validation.

### 3.5. LPS Up-Regulated miR-21 Expression and P4 Synthesis in GCs

**Figure 5:**
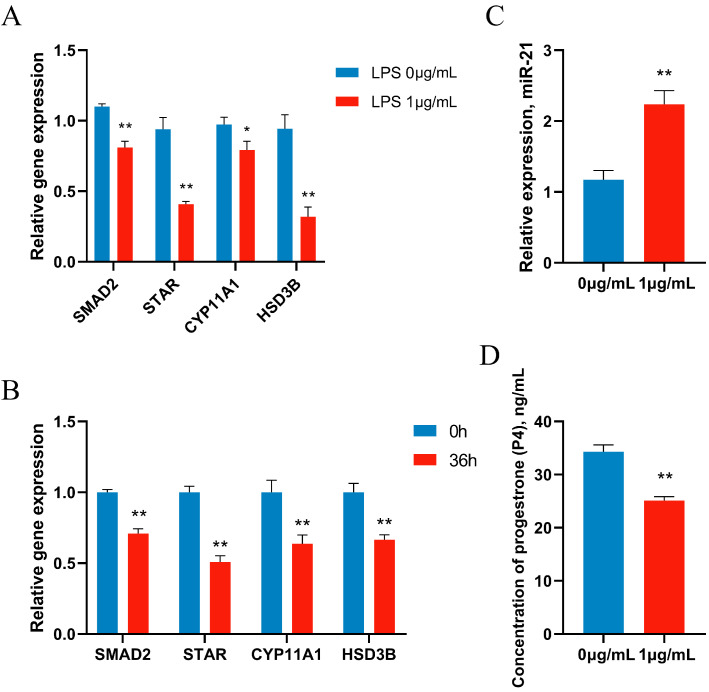
LPS inhibits progesterone synthase expressions and progesterone production. (**A**) Geese were injected with LPS (1.5 mg/kg BW) for 36 h, and progesterone synthesis-related gene expressions were examined. GCs were treated for 12 h with vehicle control or 1 μg/mL of LPS, and mRNA levels (**B**) and miR-21 level (**C**) were examined by qRT-PCR. (**D**) Progesterone (P4) levels were examined by ELISA. Data are presented as mean ± SEM, *n* = 9. *, *p* < 0.05; **, *p* < 0.01.

In animal experiments, the expression of P4-related genes in ovarian function were determined. The experimental results showed that LPS significantly down-regulated the expression of SMAD2 and P4 production-related genes STAR, CYP11A1, and HSD3B1 (Figure 5A). Next, primary GCs were treated with 1 μg/mL of LPS, and gene expression was reduced after 12 h of LPS treatment, showing the same results as in vitro experiments (Figure 5B). Accordingly, the secretion of P4 was significantly decreased after LPS treatment, and the concentration of P4 in the supernatant of the cell culture medium was significantly decreased by ELISA (Figure 5C). Meanwhile, LPS significantly increased the expression of miR-21 in vitro, further confirming the sequencing results (Figure 5D).

### 3.6. miR-21 Was Involved in LPS Regulation of P4 Synthesis in GCs

**Figure 6:**
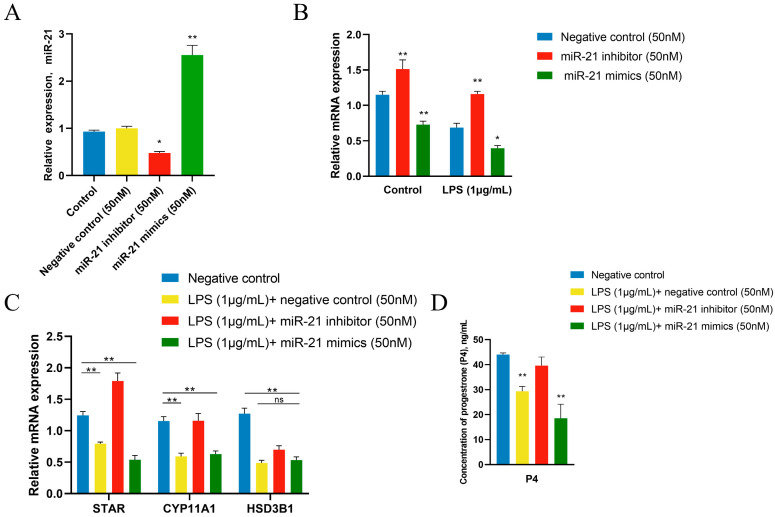
miR-21 regulates steroid secretion from GCs of the hierarchical follicles. GCs were treated for 12 h with vehicle control or 1 μg/mL of LPS after 36 h transfection with miR-21 mimics or inhibitor. (**A**) Comparison of miR-21 transfection efficiency in GCs. (**B**) The SMAD2 expression in LPS-treated GCs after transfection. (**C**) The mRNA expression of key genes related to steroid synthesis in GCs after transfection. (**D**) The concentrations of P4 in GCs after transfection. Data are presented as mean ± SEM, *n* = 9. *, *p* < 0.05; **, *p* < 0.01. ns, not significant.

To further confirm the involvement of miR-21 in LPS-induced down-regulation of P4 production, a transfection was performed to suppress and overexpress miR-21. In general, the negative control (NC) had no effect on miR-21 expression, whereas there was significant inhibition and overexpression of miR-21 (Figure 6A). Transfected GCs with 50 nM of miR-21 inhibitor significantly up-regulated SMAD2 mRNA levels, and transfected GCs with 50 nM of miR-21 mimics significantly down-regulated SMAD2 mRNA levels (Figure 6B). Overexpression of miR-21 inhibited SMAD2 expression in LPS-treated GCs; inhibition of miR-21 effectively antagonized LPS treatment and restored SMAD2 to normal expression. Subsequently, the expression of steroid hormone production pathways STAR, CYP11A1, and HSD3B1 was examined. The expression of miR-21 was negatively correlated with STAR and CYP11A1 mRNA levels, but the expression of HSD3B1 was not affected (Figure 6C). The P4 concentration was significantly increased after the use of miR-21 inhibitor, while overexpression of miR-21 showed the opposite effect (Figure 6D).

### 3.7. miR-21 Was Involved in LPS Regulation of P4 Synthesis in GCs by Down-Regulating SMAD2

**Figure 7:**
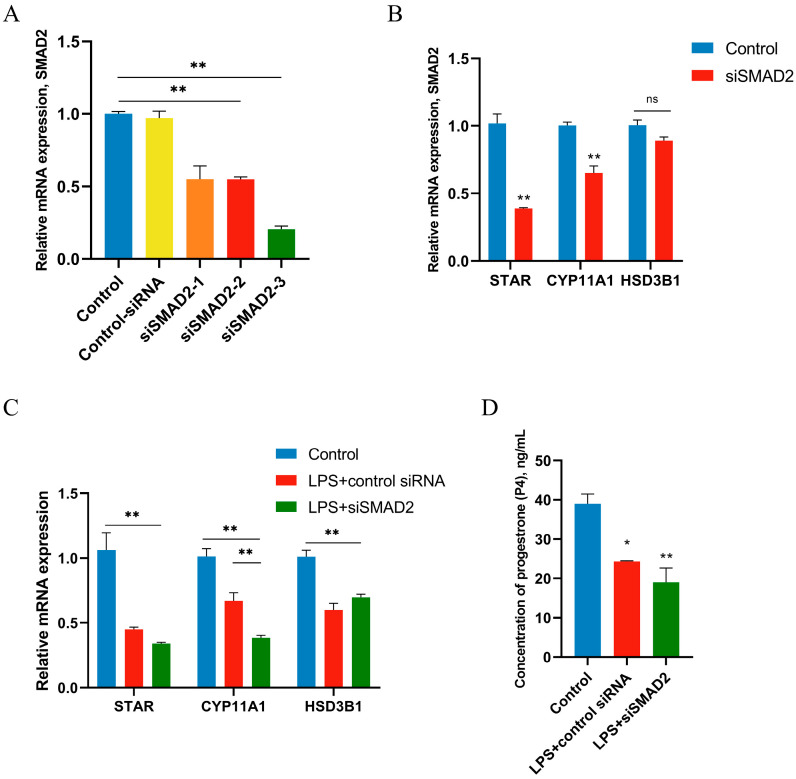
SMAD2 is necessary in P4 synthesis. GCs were treated for 12 h with vehicle control or 1 μg/mL of LPS after 36 h transfection with siRNA of SMAD2 (50 nM). (**A**) Comparison of transfection efficiency of different siSMAD2. (**B**) The mRNA expression of key genes related to steroid synthesis in GCs after transfection. (**C**) The mRNA expression of key genes related to steroid synthesis in LPS-treated GCs after transfection. (**D**) The concentrations of P4 in LPS-treated GCs after transfection. Data are presented as mean ± SEM, *n* = 9. *, *p* < 0.05; **, *p* < 0.01. ns, not significant.

Given the important role of STAR in the regulation of P4 production, the effect of SMAD2 on P4 production in GCs was next examined. A siRNA-mediated knockdown approach was used to knock down the endogenous expression of SMAD2. To figure out the role of SMAD2 in regulating P4 synthesis, siRNAs of SMAD2 were transfected into GCs. siSMAD2-3 was selected as the siRNA for this experiment by verifying the transfection efficiency of different siRNAs as the highest silencing efficiency siRNA (Figure 7A). Silencing of SMAD2 had a significant effect on STAR and CYP11A1, but the expression of HSD3B1 was not affected (Figure 7B). In LPS-treated GCs, HSD3B1 was decreased by LPS, and silencing SMAD2 did not affect HSD3B1 expression (Figure 7C). The P4 concentration was detected by ELISA, and there was no significant decrease in P4 concentration after silencing SMAD2 under LPS treatment (Figure 7D).

**Figure 8:**
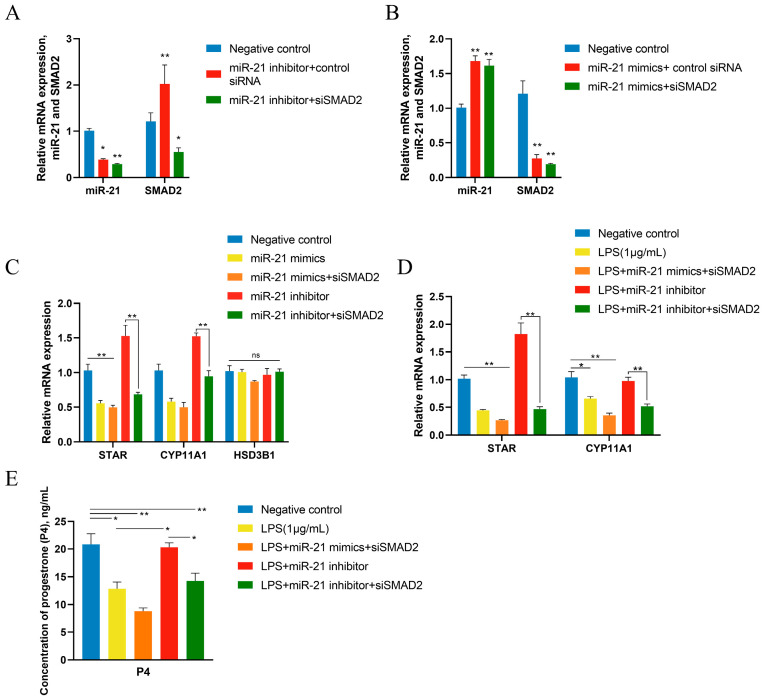
miR-21 and SMAD2 are involved in the LPS-induced decrease in progesterone secretion in GCs. GCs were treated for 12 h with vehicle control or 1 μg/mL of LPS after 36 h transfection with miR-21 mimics or inhibitors (50 nM) and siSMAD2 (50 nM). The expression of miR-21 and SMAD2 in GCs after co-transfection of miR-21 inhibitor (**A**) or miR-21 mimics (**B**) and siSMAD2. (**C**) The mRNA expression of key genes related to steroid synthesis in GCs after o-transfection. (**D**) The mRNA expression of key genes related to steroid synthesis in LPS-treated GCs after co-transfection. (**E**) The concentrations of P4 in LPS-treated GCs after transfection. Data are presented as mean ± SEM, *n* = 9. *, *p* < 0.05; **, *p* < 0.01. ns, not significant.

**Figure 9:**
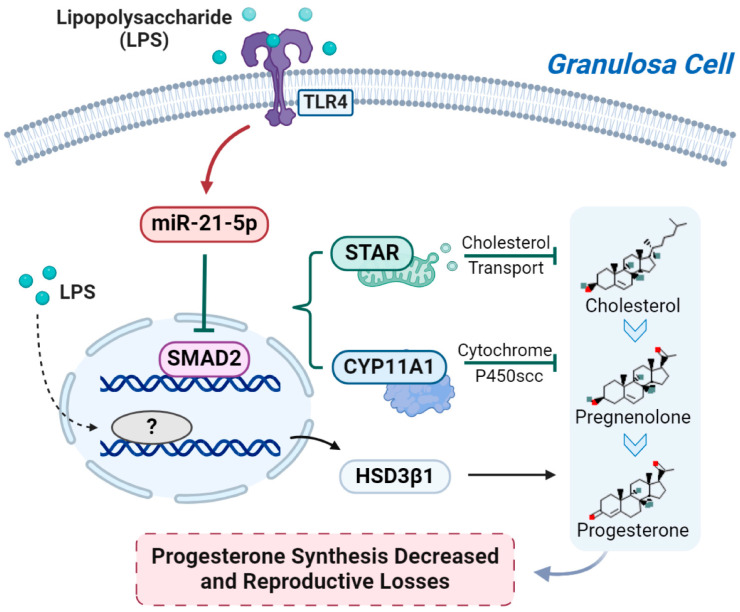
The miR-21/SMAD2 pathway is involved in LPS inhibit progesterone (P4) production in granulosa cells (GCs). The question mark represent currently unknown LPS-interacting genes.

GCs were treated for 12 h with 1 μg/mL of LPS after 36 h of transfection with miR-21 mimics (50 nM), miR-21 inhibitors (50 nM), and siSMAD2 (50 nM). After transfection with miR-21 inhibitor and siSMAD2, the expression level of miR-21 was not further decreased, while SMAD2 mRNA levels were not increased by miR-21 inhibitor, indicating that SMAD2 plays a role in the downstream of miR-21 (Figure 8A). Additionally, after transfection with miR-21 mimics and siSMAD2, the expression of miR-21 increased while SMAD2 mRNA levels decreased, which was the same as the previous experimental results (Figure 8B). Meanwhile, HSD3B1 mRNA level was not affected (Figure 8C). In LPS-treated GCs, the miR-21 mimics and siSMAD2 group caused a significant decrease in CYP11A1 (Figure 8D). Finally, miR-21 inhibitors could restore the decrease in P4 concentration caused by LPS, while siSMAD2 had a negative effect on the P4 production (Figure 8E). Collectively, miR-21 was involved in the process of LPS inhibiting P4 production (Figure 9).

## 4. Discussion

The quality of the water has a direct impact on the reproductive performance of geese. Research has shown that plasma LPS concentration can significantly depress reproductive performance [32]. LPS impairs ovarian function, granulosa cells (GCs) necrosis and apoptosis, and steroid hormone secretion through the p38, NF-κB, and MAPK pathways [33,34,35,36]. Plasma LPS will be higher in women with polycystic ovary syndrome (PCOS) [37]. miR-873-5p/HO-1 is involved in oxidative stress induced by LPS in PCOS [38]. In addition, ROS inhibits STAR expression through the GATA4/6 pathway in LPS-treated porcine granulosa-lutein cells [19]. The above results prove that the study of LPS in mammals is a comprehensive explanation of the role of LPS. In this research, geese follicles underwent gelatinous degeneration after LPS injection, the follicle shape became irregularly rounded, and it was difficult to separate the membranous layer, granulosa layer, and yolk, proving that the degenerated follicles had lost the follicular function and entered the stage of follicular atresia in early. Further, LPS-treated degenerated follicles from Yangzhou geese at the peak of egg-laying were selected as the sequencing model, and healthy follicles were used as the control group. The obtained sequencing data were analyzed and filtered, and the targeting relationship of highly differentially expressed miRNAs and mRNAs was further predicted by bioinformatics methods. In brief, miR-21 and its targeted gene SMAD2 were predicted in an attempt to investigate its molecular mechanism.

P4 is the major steroid that plays a critical role in regulating ovulation in hierarchical follicles [39]. STAR is a key gene in the secretion of all follicular steroid hormones. GCs of hierarchical follicles are stimulated by follicle-stimulating hormone (FSH) to synthesize P4 in response to STAR, CYP11A1, and HSD3B1 [40,41]. Furthermore, P4 serves as a precursor for estradiol (E2) synthesis in Theca cells (TCs) of hierarchical follicles, which confidently regulates steroid production and secretion in TCs [42]. Therefore, in this experiment, ELISA was used to detect P4 secretion, while qPCR was used to measure the expression of STAR, CYP11A1, and HSD3B1. The effect of LPS exposure on P4 production was verified by in vivo and in vitro experiments. In animal experiments, a decrease in serum P4 concentration was detected in Yangzhou geese at breeding stage after LPS injection. The P4 level decreased significantly after injection of 1.5 mg/kg of LPS for 24 h, and P4 decreased further after 36 h LPS injected. To further verify the effect of LPS on progesterone secretion in granulosa cells, primary GCs were treated with LPS, and the assay revealed that treatment of GCs with 1 μg/mL of LPS for 12 h inhibited progesterone production and down-regulated the STAR, CYP11A1, and HSD3B1 mRNA levels. The experimental results indicated that LPS had a negative effect on P4 and P4-related genes.

As a non-coding RNA, miR-21 has an initial sequence that is conserved across multiple species, and existing studies have shown that miR-21 is extensively involved in physiological and pathological processes of the reproductive system.

miR-21 has a known regulatory function in human reproduction-related diseases such as breast cancer, ovarian cancer, and endometriosis. miR-21 may inhibit the growth and metastasis of luminal-like breast cancer by inhibiting the NF-κB/PDCD4 axis [43]. The exosome miR-21 promotes ovarian cancer progression by regulating CDK6 [44]. Up-regulation of YAP1/miR-21-5p decreases PGR expression and plays a negative role in the development of endometriosis [45]. In addition, another report found that miR-21 in GCs regulates the expression of STAR through down-regulation of LATS1, which ultimately promotes the secretion of estrogen in GCs [46]. Duan et al. found that miR-21 down-regulation is involved in infection-induced preterm birth (PTB), contributing to LPS through the NF-κB pathway, which is the only study on the modulation of miR-21 by LPS resulting in reproduction-related system diseases [47]. The above findings suggest a potential role for miR-21 in ovarian function regulation, but the role in avian GCs requires further exploration. In this study, transcriptome analysis showed that 51 miRNAs were abnormally expressed in the follicular granulosa layer exposed to LPS, including 20 down-regulated miRNAs and 31 up-regulated miRNAs. Through bioinformatics prediction and in vitro analysis, it was found that the down-regulation of SMAD2 mediated by miR-21 inhibited P4 synthesis in GCs and affected the ovulation process under the action of LPS. As miR-21 was confirmed to be enriched in steroid synthesis pathways, this study explored the specific role of miR-21 in steroid synthesis in GCs. The findings indicated that transfection with miR-21 mimics or inhibitors had a significant effect on P4 synthesis. Overexpression of miR-21 inhibited sex steroid biosynthesis by suppressing the expression of SMAD2 and decreasing the expression of STAR and CYP11A1, decreasing P4 secretion while having no effect on the expression of HSD3B1. Up-regulation of miR-21 exacerbated the LPS-induced decline in P4 synthesis and vice versa. These findings are consistent with previous studies that have validated the crucial role of miRNAs in the synthesis of steroidal GCs [27,48].

The SMAD (mothers against decapentaplegic homolog) family is an important new gene family in the transforming growth factor beta (TGF-β) superfamily. Multiple studies have shown that the TGF-β/SMAD pathway is involved in mammalian follicular development and hormone secretion [49,50]. LPS/TLR4 is also involved in a number of pathological processes by affecting the TGF-β pathway [51,52]. TGF-β/SMAD2/KLF8 axis regulates epithelial–mesenchymal transition (EMT) and contributes to ovarian cancer progression. The TGFBR2/Smad2 signaling pathway has a critical role in LPS-induced sepsis [53]. SMAD2 was shown to function as a transcription factor for STAR. Activation of SMAD1/5/8 and SMAD2/3 signaling pathways was found to down-regulate the expression of STAR and inhibit P4 synthesis in human GCs [54]. TGF-1 affects estradiol and P4 synthesis in goat GCs through the SMAD/SP1 pathway [55]. These results suggest that SMAD2 may play a regulatory role in animal ovarian function. Our study further investigated the effects of silencing SMAD2 on GCs. Silencing SMAD2 has a synergistic effect in LPS-treated GCs. Furthermore, SMAD2 also inhibits P4 secretion in GCs by decreasing the expression of STAR and CYP11A1. At the same time, after the appropriate concentration was determined through pre-experiments, miR-21 mimics, inhibitors, and siRNA of SMAD2 were transfected into GCs together. It was found that silencing SMAD2 had no significant effect on the expression of miR-21, indicating that SMAD2 mainly plays a regulatory role in the downstream of miR-21. In addition, HSD3B1 was not affected by changes in miR-21 but was decreased by LPS, suggesting that it may be regulated by other undiscovered pathways. Interestingly, it was found that miR-21 targets STARD9 (steroid transport protein domain 9), which has been shown to be involved in cholesterol transport. This indicates that miR-21 may be the key miRNA of various sex hormones in GCs. It should be noted that the functions of P4 in mammals and birds are not the same; further studies are needed on the regulatory pathway of granulosa cell P4 production in birds and the effects of miRNA on follicle hormone secretion at other developmental stages. These data indicated that up-regulated miR-21 inhibits SMAD2 expression and was involved in the LPS-induced reduction of P4 production in GCs.

## 5. Conclusions

In summary, in this study, miRNA-seq was performed in LPS-exposed Yangzhou geese at peak egg-laying period, and then an LPS injury model of primary GCs was established. miR-21 was found to inhibit P4 secretion in LPS-injured GCs of Yangzhou geese by suppressing SMAD2 expression. At the miRNA level, it was further verified that miR-21 regulates SMAD2 and participates in the important mechanism of LPS injury reproduction function by gene knockdown. The results of this research will be used as a tool for understanding the miRNA regulatory network, to clarify the molecular mechanism of LPS regulation of hierarchical follicle development and hormone secretion, and to provide a more in-depth solution to LPS pollution. Our results may provide future scientific insights into miRNA regulation in LPS-damaged GCs in the future.

## Figures and Tables

**Table 1:** Highly expressed DEMs between HF and DF.

#ID	HF (Mean TPM)	DF (Mean TPM)	*p* Value	Log2FC	Regulated
aca-miR-145-5p	4.69	1533.23	<0.001	8.147	up
aca-miR-375-3p	500.16	2.4	<0.001	−7.954	down
aca-miR-145-3p	2.29	330.66	<0.001	6.963	up
aca-miR-143-3p	237.78	33,591.19	<0.001	6.947	up
aca-miR-199a-5p	19.68	1249.16	<0.001	5.7	up
aca-miR-21	1242.66	46,442.46	<0.001	4.986	up
aca-miR-1388-5p	14.67	522.74	<0.001	4.913	up
aca-miR-204a-5p	228.54	9.11	<0.001	−4.886	down
aca-miR-205a	6.03	173.2	<0.001	4.637	up
aca-miR-122-5p	570.11	33.26	<0.001	−4.24	down

## Data Availability

The original contributions presented in the study are included in the article/Supplementary Materials, further inquiries can be directed to the corresponding author due to privacy.

## Supplementary Materials

The following supporting information can be downloaded at: https://www.mdpi.com/article/10.3390/metabo14070362/s1, Figure S1: GO analysis of differentially expressed miRNA target genes; Table S1: Primers used for quantitative real-time PCR; Table S2: Summary statistics of the transcriptome sequencing for geese from HF and DF; Table S3: Predict target genes of DEMs between HF and DF.

## Abbreviations

LPS	Lipopolysaccharide
P4	Progesterone
GCs	Granulosa Cells
SMAD2	SMAD family member 2
STAR	steroidogenic acute regulatory protein
CYP11A1	cytochrome P450 family 11 subfamily A member 1
HSD3B1	3β-hydroxysteroid dehydrogenase
HF	GCs of the control group hierarchical follicles
DF	GCs of the LPS treated denatured hierarchical follicles
