# Supplementary material for: miR-21/SMAD2 Is Involved in the Decrease in Progesterone Synthesis Caused by Lipopolysaccharide Exposure in Follicular Granulosa Cells of Laying Goose
Source: Metabolites. 2024 Jun 27;14(7):362. doi: 10.3390/metabo14070362 (PMC11278936; doi:10.3390/metabo14070362)
Supplement: Supplementary file 1 [file metabolites-14-00362-s001.zip › Supplementary_Material.pdf]

Supplementary Material

1 Supplementary Data

Figure S1. GO analysis of differentially expressed miRNA target genes.

Table S1. Primers used for quantitative real-time PCR

Table S2. Summary statistics of the transcriptome sequencing for geese from HF and DF

Table S3. Predict target genes of DEMs between HF and DF

2 Supplementary Figures and Tables

2.1 Supplementary Figures

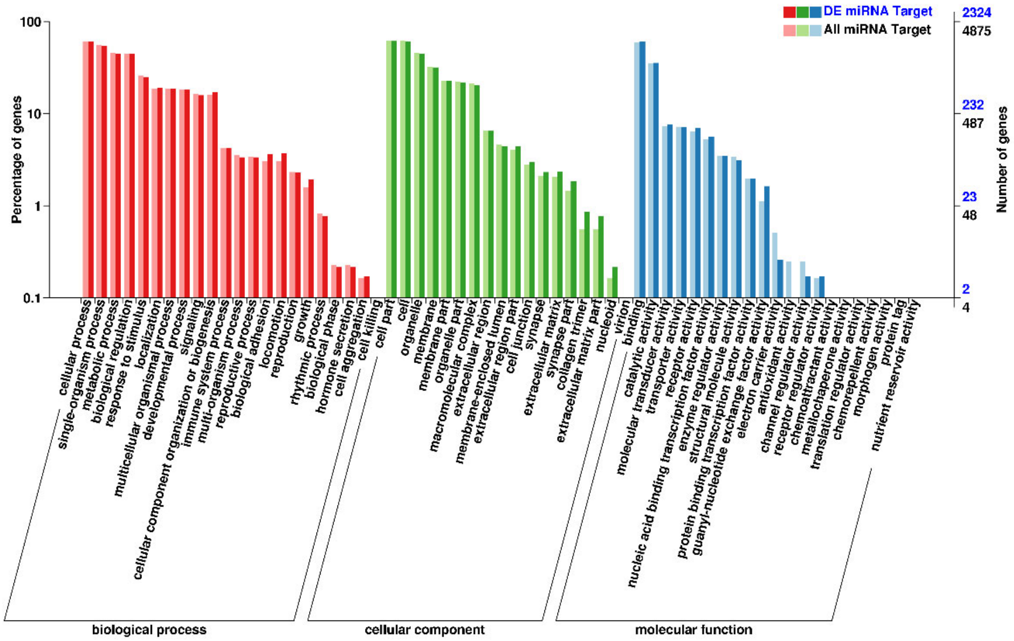

Figure S2. GO analysis of differentially expressed miRNA target genes.

2.2 Supplementary Table

Table S1. Primers used for quantitative real-time PCR

Gene	Primer Sequences (5' to 3')	Annealing	Length	Accession number
β-actin	F: CGAATCCGGACCCTCCATTG<br>R: TTTGTCACAAGGGTGTGGGT	60℃	77bp	XM_013174886.1
STAR	F: GTGCGGTGGAAAGCGAATTT<br>R: CGGGTGCTCGGCCTTAAATA	60℃	145bp	XM_013194444.2
CYP11A1	F: TGATCCTTCCCCCAGACACT<br>R: GAAGCTCAGCCCCTTGAAGT	60℃	138bp	XM_048081522.1
HSD3B1	F: TGTAGCAAGGGAGACGTGAG<br>R: CTTGGTTCCACCCCTCTAGC	60℃	119bp	XM_013196414.2
SMAD2	F: TGGACCTGGGGGCATTGAT<br>R: CGTTCAGGTTTTCCGCCTCG	60℃	81bp	XM_048054491.1
miR-21a-5p	F: GCGGTAGCTTATCAGACTGATGTTGA<br>R: CAGGTCCAGTTTTTTTTTTTTTT	60℃	\	MIMAT0021865 (miRBase)
U6	F: GGGCCATGCTAATCTTCTCTGTA<br>R: CAGGTCCAGTTTTTTTTTTTTTT	60℃	\	\

Table S2. Summary statistics of the transcriptome sequencing for geese from HF and DF

**Samples**	**Raw_reads**	**Clean_reads**	**Mapped_Reads**	**Q30(%)**
HF_1	20024465	18424497	12818614	98.80
HF_2	23685341	22419694	15684811	98.95
HF_3	30282574	23978828	10411619	98.66
DF_1	22094213	21274748	13058267	98.70
DF_2	22509993	21479759	12653668	98.56
DF_3	17093597	15942353	9058793	98.77

Table S3. Predict target genes of DEMs between HF and DF

miRNA_ID	Gene_ID
aca-miR-122-5p	MDK
aca-miR-1388-5p	MFSD7
aca-miR-1388-5p	ZFYVE9
aca-miR-143-3p	AGPAT4
aca-miR-143-3p	UBAP2
aca-miR-143-3p	SLC26A8
aca-miR-145-3p	PAMR1
aca-miR-145-5p	EPS8L2
aca-miR-145-5p	CCDC50
aca-miR-145-5p	LOC106048078
aca-miR-145-5p	NRP2
aca-miR-145-5p	DNTTIP2
aca-miR-145-5p	RIMS4
aca-miR-145-5p	CACNA2D3
aca-miR-145-5p	ARHGAP32
aca-miR-145-5p	BMP7
aca-miR-145-5p	SLC26A3
aca-miR-145-5p	SFMBT1
aca-miR-145-5p	ADAMTSL3
aca-miR-145-5p	TAGAP
aca-miR-145-5p	ACSS1
aca-miR-145-5p	SPIN1
aca-miR-145-5p	GRTP1
aca-miR-145-5p	ANKRD11
aca-miR-145-5p	CUEDC2
aca-miR-145-5p	SLC35F1
aca-miR-145-5p	STOML2
aca-miR-145-5p	PCSK1
aca-miR-145-5p	STRIP2
aca-miR-145-5p	LOC106049530
aca-miR-145-5p	SLC35B2
aca-miR-145-5p	LOC106049641
aca-miR-145-5p	TNXB
aca-miR-145-5p	AHNAK
aca-miR-145-5p	COL19A1
aca-miR-145-5p	FAM60A
aca-miR-145-5p	MAPK1
aca-miR-145-5p	XDH
aca-miR-145-5p	LOC106033173
aca-miR-145-5p	COL24A1
aca-miR-145-5p	ANGPTL5

aca-miR-145-5p	COL3A1
aca-miR-145-5p	EPSTI1
aca-miR-145-5p	ELF1
aca-miR-145-5p	MDN1
aca-miR-145-5p	CHL1
aca-miR-145-5p	COL4A3
aca-miR-145-5p	COL4A4
aca-miR-145-5p	EPHA4
aca-miR-145-5p	CCBE1
aca-miR-145-5p	MAATS1
aca-miR-145-5p	ANO4
aca-miR-145-5p	MYO5B
aca-miR-199a-5p	PRSS12
aca-miR-199a-5p	ARHGAP29
aca-miR-199a-5p	SRI
aca-miR-199a-5p	CCDC88A
aca-miR-199a-5p	INPP5F
aca-miR-199a-5p	RAB30
aca-miR-199a-5p	PALM
aca-miR-199a-5p	PDIA5
aca-miR-199a-5p	PLEKHH1
aca-miR-199a-5p	NRK
aca-miR-204a-5p	COL6A2
aca-miR-204a-5p	NRP2
aca-miR-204a-5p	PLCE1
aca-miR-204a-5p	PODXL
aca-miR-204a-5p	LOC106039917
aca-miR-204a-5p	BLVRA
aca-miR-204a-5p	SPTLC1
aca-miR-204a-5p	FLNC
aca-miR-204a-5p	PDGFRB
aca-miR-204a-5p	CHAF1B
aca-miR-204a-5p	THSD7A
aca-miR-205a	LOC106047013
aca-miR-205a	LOC106048078
aca-miR-205a	COL6A2
aca-miR-205a	LOC106049840
aca-miR-205a	SH3BP1
aca-miR-205a	LOC106038569
aca-miR-205a	KLF3
aca-miR-205a	EPHB1
aca-miR-205a	CD2
aca-miR-205a	LOC106039827
aca-miR-205a	IARS

aca-miR-205a	PCDH18
aca-miR-205a	CLIP2
aca-miR-205a	ZNF217
aca-miR-205a	IKBKE
aca-miR-205a	DYSF
aca-miR-205a	ERICH3
aca-miR-205a	AKAP1
aca-miR-205a	SLC4A4
aca-miR-205a	H6PD
aca-miR-205a	PLBD1
aca-miR-205a	USP49
aca-miR-205a	PRKCB
aca-miR-205a	LMOD1
aca-miR-205a	LOC106046278
aca-miR-205a	GPATCH8
aca-miR-205a	ZYX
aca-miR-205a	LOC106048077
aca-miR-205a	ADAMTS10
aca-miR-205a	ESCO2
aca-miR-205a	SCARA5
aca-miR-205a	LOC106049539
aca-miR-205a	LRRC3C
aca-miR-205a	TUFT1
aca-miR-205a	LOC106049857
aca-miR-205a	PRF1
aca-miR-205a	SLC15A2
aca-miR-205a	TTN
aca-miR-205a	ABI2
aca-miR-205a	CDH23
aca-miR-205a	DENND5B
aca-miR-205a	LOC106031105
aca-miR-205a	RYR3
aca-miR-205a	LOC106032300
aca-miR-205a	LOC106032484
aca-miR-205a	MYB
aca-miR-205a	MAF
aca-miR-205a	XYLT1
aca-miR-205a	AGAP1
aca-miR-205a	ITGB4
aca-miR-205a	TRIM13
aca-miR-205a	TLN1
aca-miR-205a	LOC106036638
aca-miR-205a	CCDC13
aca-miR-205a	GPLD1

aca-miR-205a	FYCO1
aca-miR-375-3p	LOC106049840
aca-miR-375-3p	ADAMTS7
aca-miR-375-3p	UBXN6
aca-miR-375-3p	ADAMTS2
aca-miR-375-3p	RNF103
aca-miR-375-3p	RAB11FIP3
aca-miR-375-3p	CTNND1
aca-miR-375-3p	RELN
aca-miR-21-5p	STARD9
aca-miR-21-5p	LRP2
aca-miR-21-5p	ABCF3
aca-miR-21-5p	SMAD2
aca-miR-21-5p	STAR
aca-miR-21-5p	FAM184B
