# Supplementary figures and images for: miR-21/SMAD2 Is Involved in the Decrease in Progesterone Synthesis Caused by Lipopolysaccharide Exposure in Follicular Granulosa Cells of Laying Goose
Source: Metabolites. 2024 Jun 27;14(7):362. doi: 10.3390/metabo14070362 (PMC11278936; doi:10.3390/metabo14070362)

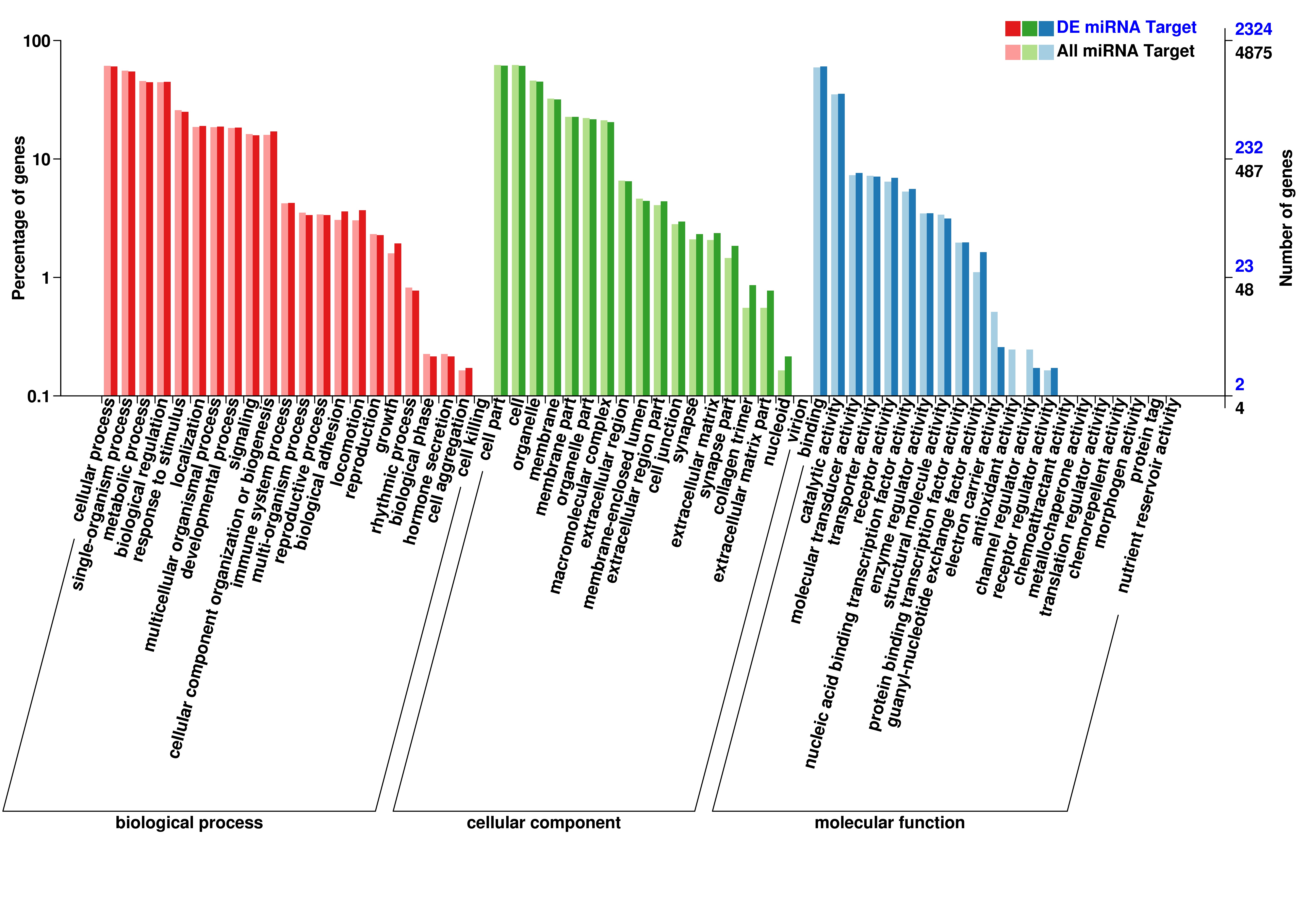

Supplement: Supplementary file 1 [file metabolites-14-00362-s001.zip › Supplementary Figure S1. GO analysis of differentially expressed miRNA target genes..jpg]
